# Morphometric analysis and spatial localization of the infraorbital foramen in adult dry human skulls: bilateral symmetry, sexual dimorphism, and accessory foramina

**DOI:** 10.3389/fmed.2026.1914818

**Published:** 2026-08-05

**Authors:** Figen Koç Direk, Ayşe Acar

**Affiliations:** 1 Department of Anatomy, Faculty of Medicine, Mardin Artuklu University, Mardin, Türkiye; 2 Department of Anthropology, Faculty of Letters, Mardin Artuklu University, Mardin, Türkiye

**Keywords:** accessory infraorbital foramen, craniofacial anatomy, infraorbital foramen, infraorbital nerve block, maxillofacial surgery, morphometry, orbital reconstruction, sexual dimorphism

## Abstract

**Background:**

The infraorbital foramen (IOF) is a key anatomical landmark for infraorbital nerve block, maxillofacial surgery, and orbital reconstruction. However, its location and morphology exhibit considerable individual and population-based variation, emphasizing the need for population-specific morphometric data.

**Methods:**

This anatomical study evaluated 103 adult dry human skulls (206 hemimaxillae). Bilateral measurements of IOF coordinates, dimensions, and distances to adjacent anatomical landmarks, craniofacial measurements (bizygomatic width and upper facial height), IOF shape and orientation, and the presence of accessory infraorbital foramina (AIOF) were obtained. Morphometric indices, including the lateralization index, vertical position index, and alveolo-orbital ratio, were calculated. Bilateral and sex-related differences were analyzed using appropriate parametric statistical tests. Correlation analyses were performed to investigate relationships between IOF morphology and craniofacial dimensions. Spatial distribution of the IOF was evaluated using kernel density estimation, and receiver operating characteristic (ROC) analysis was performed to assess morphometric predictors of AIOF.

**Results:**

Significant bilateral differences were observed for horizontal and vertical IOF coordinates, lateralization index, and vertical position index, which were statistically different between sides, whereas foraminal diameters, IOF–infraorbital margin distance, IOF–alveolar crest distance, and alveolo-orbital ratio showed no significant side differences. Male skulls demonstrated significantly greater bizygomatic width, upper facial height, and horizontal and vertical IOF coordinates, whereas females exhibited significantly higher vertical position indices, with all these parameters being statistically different between sexes. Strong positive correlations were identified between craniofacial dimensions and IOF localization. AIOFs were identified in 18.4% of hemimaxillae without significant side predominance. Skulls with AIOFs exhibited significantly smaller vertical IOF diameters, shorter IOF–infraorbital margin distances, and higher alveolo-orbital ratios. ROC analysis demonstrated excellent predictive performance for mean vertical IOF diameter (AUC = 0.995) and mean IOF–infraorbital margin distance (AUC = 0.950).

**Conclusion:**

The infraorbital foramen demonstrates subtle bilateral asymmetry and significant sex-related variation that reflect overall craniofacial morphology. The integration of morphometric indices, spatial density mapping, and AIOF-related analyses provides a comprehensive framework for anatomical assessment of the infraorbital region. These findings offer clinically relevant reference data for infraorbital nerve block, maxillofacial surgery, orbital reconstruction, and related procedures involving the midface.

## 1 Introduction

The infraorbital foramen (IOF), located on the anterior surface of the maxilla, represents the terminal opening of the infraorbital canal and serves as the exit point for the infraorbital nerve and accompanying vessels (1). As a continuation of the maxillary division of the trigeminal nerve, the infraorbital nerve provides sensory innervation to the lower eyelid, lateral aspect of the nose, upper lip, and adjacent facial soft tissues. Consequently, precise localization of the IOF is of considerable importance in regional anesthesia, maxillofacial surgery, orbital reconstruction, trauma management, and esthetic facial procedures (2, 3).

Accurate identification of the IOF is essential for successful infraorbital nerve block and for minimizing iatrogenic neurovascular injury during surgical interventions involving the midface (4). Variations in the location, dimensions, morphology, and direction of the IOF, as well as the presence of accessory infraorbital foramina, may contribute to anesthetic failure or unexpected surgical complications (4). Therefore, detailed anatomical knowledge of these variations is critical for clinicians involved in oral and maxillofacial surgery, plastic surgery, otolaryngology, and interventional pain management (5).

Previous morphometric investigations have demonstrated substantial population-specific variability in IOF characteristics, including its relationship to surrounding osseous landmarks, foraminal dimensions, and accessory foramina prevalence (4, 6). Furthermore, sex-related differences and bilateral asymmetry have been reported inconsistently among different ethnic populations, highlighting the need for population-specific reference data (7). Most contemporary studies have employed cone-beam computed tomography (CBCT), which enables high-resolution three-dimensional assessment while reducing image distortion and superimposition compared with conventional radiographic techniques (2, 8, 9).

In addition to absolute morphometric measurements, proportional indices describing the relative position of the IOF within the facial skeleton may provide clinically meaningful information by accounting for individual differences in craniofacial size (10, 11). Correlation analyses between craniofacial dimensions and IOF localization may further improve anatomical prediction models and facilitate safer surgical planning. However, studies integrating conventional morphometric measurements with positional indices and craniofacial proportional analyses remain limited (12, 13). Furthermore, although bilateral symmetry and sexual dimorphism of the IOF have been investigated in several populations, the reported findings remain inconsistent, emphasizing the need for population-specific anatomical data. Moreover, quantitative spatial mapping approaches, such as kernel density estimation and probability density contour analysis, have rarely been applied to visualize the anatomical distribution of the IOF and its accessory foramina (14, 15). Similarly, studies evaluating the diagnostic performance of morphometric parameters for predicting the presence of accessory infraorbital foramina using receiver operating characteristic (ROC) analysis remain scarce.

Therefore, the present study aimed to comprehensively evaluate the morphometric characteristics, bilateral symmetry, sexual dimorphism, accessory infraorbital foramen distribution, and anatomical variations of the infraorbital foramen in adult dry human skulls. In addition to conventional morphometric measurements, positional indices, spatial density mapping, and ROC curve analyses were used to investigate the anatomical localization and morphometric predictors of accessory infraorbital foramina. Sex-based morphometric comparisons were also performed to identify potential anatomical differences relevant to clinical practice. The findings were intended to provide clinically applicable anatomical reference data for regional anesthesia and surgical procedures involving the midfacial region.

## 2 Materials and methods

### 2.1 Study design and ethical approval

This descriptive, cross-sectional morphometric study was conducted using adult dry human skulls obtained from the osteological collections of the Bone Laboratory, Department of Anatomy, Faculty of Medicine, Mardin Artuklu University, and the Human Osteology Laboratory, Department of Anthropology, Mardin Artuklu University. The study protocol was reviewed and approved by the Non-Interventional Clinical Research Ethics Committee of Mardin Artuklu University (approval date: 25 March 2026; approval number: 2026/2–16) and was conducted in accordance with the ethical principles of the Declaration of Helsinki applicable to anatomical research involving human skeletal remains.

### 2.2 Study sample

A total of 103 adult dry human skulls with preserved maxillary anatomy were included in the study, providing 206 hemimaxillae for morphometric analysis. Each side was evaluated independently for anatomical characteristics, whereas bilateral comparisons were performed using paired analyses at the skull level. Skulls exhibiting severe trauma, fractures, congenital craniofacial anomalies, pathological bone destruction, previous surgical alterations, or marked erosion of the infraorbital region that could interfere with morphometric measurements were excluded. Only specimens with intact infraorbital margins, alveolar crests, and clearly identifiable infraorbital foramina were included.

### 2.3 Sex estimation

Sex estimation was performed using standard osteological methods based primarily on morphological characteristics of the pelvis and skull. Pelvic traits, including the greater sciatic notch, ventral arc, subpubic concavity, and medial aspect of the ischiopubic ramus, were evaluated whenever preservation permitted (16, 17). Cranial assessment included the morphology of the nuchal crest, mastoid process, supraorbital margin, glabella, and mental eminence. In cases with incomplete pelvic remains, cranial features and available long bone characteristics were used as supplementary criteria. Sex estimation was performed according to the recommendations of the Workshop of European Anthropologists and established osteological standards (17). Individuals lacking sufficient diagnostic skeletal elements were classified as indeterminate and excluded from sex-specific analyses to minimize potential misclassification.

### 2.4 Morphometric measurements

All measurements were performed bilaterally using a standardized protocol, with the center of the infraorbital foramen (IOF) used as the reference point. The recorded parameters included the horizontal (IOF_X) and vertical (IOF_Y) coordinates of the IOF, vertical and horizontal diameters, distances from the IOF to the infraorbital margin and alveolar crest, presence and location of accessory infraorbital foramina (AIOFs), IOF–AIOF distance when applicable, bizygomatic width (Zy–Zy), and upper facial height (Nasion–Anterior Nasal Spine). For bilateral analyses, mean values and side-to-side differences were calculated for paired measurements. Direct linear measurements were performed using a digital caliper (YAMAYO, Japan) with a resolution of 0.01 mm. Before each measurement session, the caliper was checked for proper zero alignment by fully closing the measuring jaws and was reset using the zero function when necessary. All measurements were performed by a single observer using a standardized measurement protocol and predefined anatomical landmarks.

**Figure 1:**
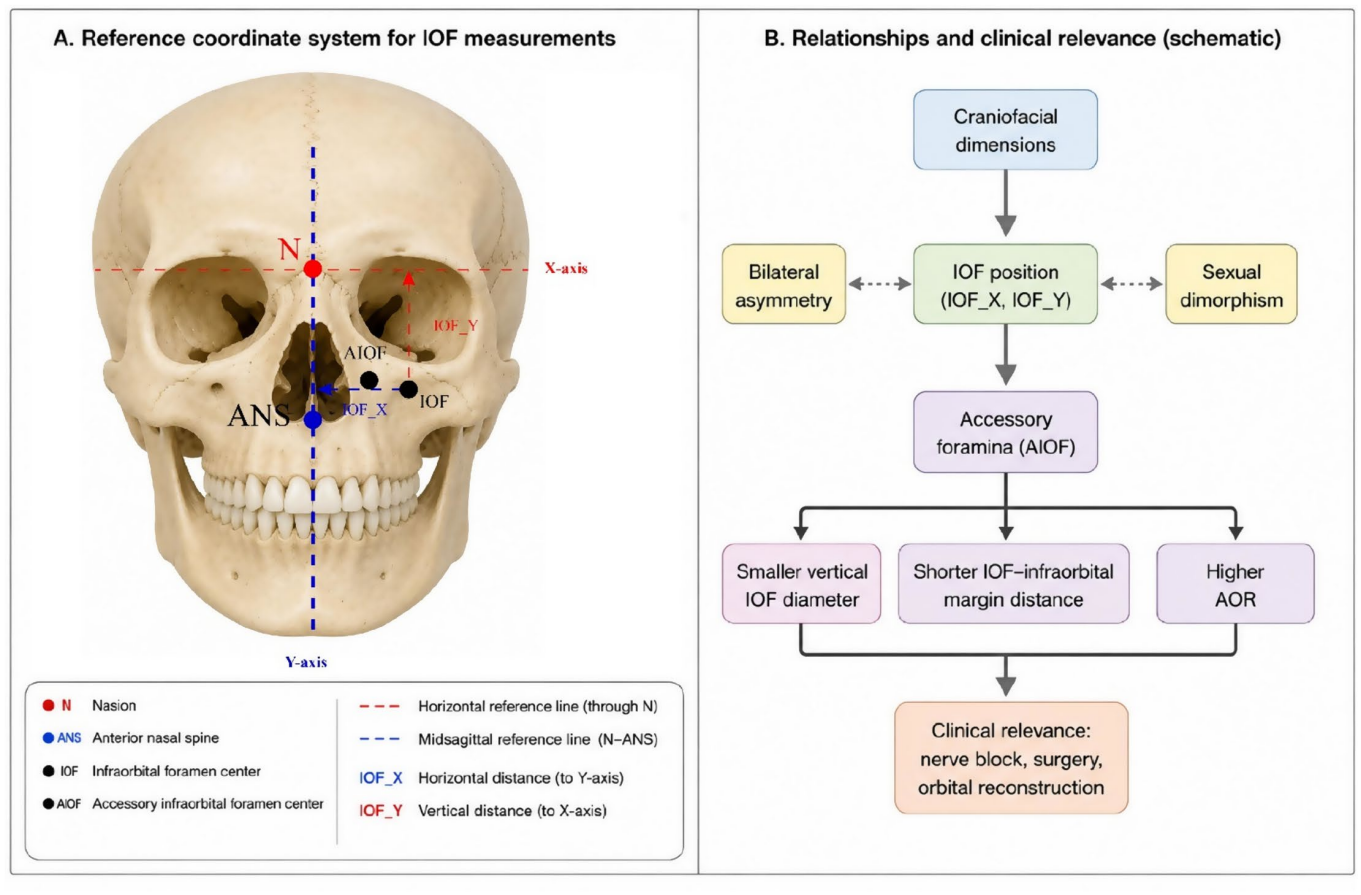
**(A)** Reference coordinate system used to determine the horizontal (IOF_X) and vertical (IOF_Y) positions of the infraorbital foramen (IOF) and accessory infraorbital foramen (AIOF). **(B)** Schematic illustration summarizing the relationships between craniofacial dimensions, IOF position, accessory foramina, and their clinical relevance.

For coordinate-based measurements, standardized frontal images were obtained with the skull positioned in the Frankfort horizontal plane. A millimetric reference scale placed within the image plane was used for spatial calibration in ImageJ. The midsagittal line passing through the nasion and anterior nasal spine was defined as the vertical reference axis, and a perpendicular horizontal line passing through the nasion served as the horizontal reference axis, with the nasion defined as the coordinate origin. IOF_X and IOF_Y were measured as the perpendicular horizontal and vertical distances, respectively, from the geometric center of the IOF to these reference axes. The same coordinate system was applied to AIOFs when present (Figure 1).

Three proportional indices were developed specifically for the present study to normalize IOF position relative to individual craniofacial dimensions and reduce the influence of absolute skull size. The lateralization index (LI) represents the horizontal position of the IOF relative to bizygomatic width, the vertical position index (VPI) expresses its vertical position relative to upper facial height, and the alveolo-orbital ratio (AOR) represents the proportional relationship between the IOF-to-alveolar crest and IOF-to-infraorbital margin distances. To assess intraobserver reliability, all measurements were repeated on 20 randomly selected skulls by the same observer after a two-week interval. The intraclass correlation coefficient demonstrated excellent agreement (ICC = 0.93). Interobserver reliability was not assessed because all measurements were performed by a single observer.

### 2.5 Derived morphometric indices

To reduce the influence of overall craniofacial size differences among specimens, three normalized morphometric indices were calculated:

- Lateralization Index (LI) = horizontal coordinate of the infraorbital foramen (IOF_X) / bizygomatic width
- Vertical Position Index (VPI) = [Upper facial height (Nasion–ANS) − IOF_Y] / Upper facial height (Nasion–ANS)
- Alveolo-Orbital Ratio (AOR) = distance from the IOF to the alveolar crest / distance from the IOF to the infraorbital margin.

These normalized parameters enabled comparison of IOF localization while minimizing the influence of interindividual differences in craniofacial size.

### 2.6 Morphological evaluation

The shape of the infraorbital foramen was classified into three morphological categories: round, oval, and vertically oval (13). The orientation of the infraorbital foramen was classified as horizontal, vertical, or oblique according to the long axis of the foramen. The presence or absence of accessory infraorbital foramina was recorded separately for each side.

### 2.7 Spatial density mapping

The spatial distribution of the infraorbital foramen and accessory infraorbital foramina was visualized using two-dimensional kernel density estimation based on IOF_X and IOF_Y coordinates. These coordinates were obtained using the standardized anatomical reference system described above, with the nasion defined as the origin, the nasion–ANS midsagittal line as the vertical reference axis, and the perpendicular line passing through the nasion as the horizontal reference axis. All coordinates were expressed in millimeters within this common anatomical reference system before density estimation. Probability density heatmaps with contour levels representing the 50, 75, and 90% distribution regions were generated to identify the most frequent anatomical locations of the foramina (4, 18).

### 2.8 Statistical analysis

Statistical analyses were performed using IBM SPSS Statistics for Windows, Version 25.0 (IBM Corp., Armonk, NY, USA). Data distribution was assessed using the Shapiro–Wilk test together with skewness and kurtosis values. Continuous variables were presented as mean ± standard deviation (SD), whereas categorical variables were expressed as frequencies and percentages. Bilateral differences between right and left sides were evaluated using paired-samples Student’s *t*-tests. Sex-related differences and comparisons according to the presence of accessory infraorbital foramina (AIOFs) were analyzed using independent-samples Student’s *t*-tests. When the assumption of homogeneity of variances was violated, Welch-corrected results were reported. The distributions of IOF shape and orientation according to sex were compared using the Pearson chi-square test. Pearson correlation analysis was performed to investigate associations between craniofacial dimensions, morphometric measurements, and positional indices. Intraobserver reliability was assessed using the intraclass correlation coefficient (ICC). Receiver operating characteristic (ROC) curve analysis was used to assess the ability of selected morphometric parameters to discriminate the presence of AIOFs. The area under the curve (AUC) and corresponding 95% confidence intervals (95% CIs) were calculated. Spatial distribution of the IOF was visualized using two-dimensional kernel density estimation (KDE) based on horizontal and vertical coordinate measurements. Probability density contours representing the 50, 75, and 90% highest-density regions (P50, P75, and P90) were superimposed, and AIOFs were plotted to demonstrate their spatial relationship with the main foramen cluster. A two-tailed *p*-value < 0.05 was considered statistically significant.

## 3 Results

### 3.1 Overall morphometric characteristics

A total of 206 hemifaces obtained from 103 adult dry skulls were included in the morphometric analysis. The mean bizygomatic width and nasion–anterior nasal spine height were 127.80 ± 3.25 mm and 69.09 ± 2.87 mm, respectively. The mean horizontal and vertical coordinates of the infraorbital foramen (IOF) were 25.88 ± 1.32 mm and 33.71 ± 1.86 mm, respectively. The mean vertical and horizontal diameters of the IOF were 3.27 ± 0.48 mm and 3.49 ± 0.24 mm, respectively. The average distances from the IOF to the infraorbital margin and alveolar crest were 7.22 ± 0.90 mm and 30.75 ± 1.91 mm, respectively. The calculated lateralization index, vertical position index, and alveolo-orbital ratio were 0.202 ± 0.007, 0.512 ± 0.015, and 4.33 ± 0.60, respectively (Table 1).

**Table 1:** Descriptive morphometric measurements of the infraorbital region (*n* = 206 hemifaces).

Variable	Mean ± SD	Min–Max
Bizygomatic width (mm)	127.80 ± 3.25	122.00–133.22
Nasion–ANS height (mm)	69.09 ± 2.87	64.30–75.03
IOF horizontal coordinate (mm)	25.88 ± 1.32	23.30–28.50
IOF vertical coordinate (mm)	33.71 ± 1.86	30.42–37.00
IOF vertical diameter (mm)	3.27 ± 0.48	1.84–3.90
IOF horizontal diameter (mm)	3.49 ± 0.24	3.01–3.92
IOF–infraorbital margin distance (mm)	7.22 ± 0.90	5.22–8.72
IOF–alveolar crest distance (mm)	30.75 ± 1.91	26.60–34.00
Lateralization index	0.2025 ± 0.0073	0.1846–0.2207
Vertical position index	0.5121 ± 0.0151	0.4648–0.5474
Alveolo-orbital ratio	4.3266 ± 0.6023	3.2804–6.2571

### 3.2 Morphological characteristics

Morphological evaluation demonstrated an equal distribution between the right and left sides (50.0% each). Of the examined hemifaces, 53.4% exhibited a round IOF, 34.5% an oval IOF, and 12.1% a vertically oval IOF. Regarding orientation, horizontal and vertical directions each accounted for 36.4%, whereas 27.2% of foramina showed an oblique orientation. An accessory infraorbital foramen (AIOF) was identified in 38 of 206 hemifaces (18.4%), while 168 hemifaces (81.6%) lacked an accessory foramen (Table 2).

**Table 2:** Morphological characteristics of the infraorbital foramen (*n* = 206 hemifaces).

Variable		*n* (%)
Side	Right	103 (50.0)
	Left	103 (50.0)
Sex	Male	98 (47.6)
	Female	108 (52.4)
IOF shape	Round	110 (53.4)
	Oval	71 (34.5)
	Vertically oval	25 (12.1)
IOF orientation	Horizontal	75 (36.4)
	Vertical	75 (36.4)
	Oblique	56 (27.2)
Accessory infraorbital foramen	Present	38 (18.4)
	Absent	168 (81.6)

The distribution of IOF morphological characteristics according to sex is presented in Table 3. Round morphology was the most frequent shape in both males (50.0%) and females (56.5%). No significant sex-related differences were observed in IOF shape (*p* = 0.549) or orientation (*p* = 0.711).

**Table 3:** Distribution of infraorbital foramen morphological characteristics according to sex.

Variable		Overall, *n* (%)	Male, *n* (%)	Female, *n* (%)	*p*-value
IOF shape	Round	110 (53.4)	49 (50.0)	61 (56.5)	0.549
	Oval	71 (34.5)	35 (35.7)	36 (33.3)	
	Vertically oval	25 (12.1)	14 (14.3)	11 (10.2)	
IOF orientation	Horizontal	75 (36.4)	38 (38.8)	37 (34.3)	0.711
	Vertical	75 (36.4)	33 (33.7)	42 (38.9)	
	Oblique	56 (27.2)	27 (27.6)	29 (26.9)	

### 3.3 Bilateral comparison

Comparison of bilateral morphometric measurements revealed statistically significant side-related differences in several parameters. The horizontal coordinate of the IOF was significantly greater on the right side than on the left (26.01 ± 1.34 mm vs. 25.75 ± 1.29 mm, *p* = 0.018). Likewise, the vertical coordinate was significantly greater on the right (33.86 ± 1.78 mm vs. 33.56 ± 1.94 mm, *p* = 0.003). The lateralization index was also significantly higher on the right side (0.2035 ± 0.0075 vs. 0.2014 ± 0.0070, *p* = 0.018), whereas the vertical position index was significantly greater on the left (0.5144 ± 0.0152 vs. 0.5099 ± 0.0147, *p* = 0.003). No significant bilateral differences were observed in IOF vertical diameter, horizontal diameter, IOF–infraorbital margin distance, IOF–alveolar crest distance, or alveolo-orbital ratio (all *p* > 0.05) (Table 4).

**Table 4:** Comparison of right and left infraorbital foramina (paired analysis, *n* = 103 skulls).

Parameter	Right (Mean ± SD)	Left (Mean ± SD)	*p* value
IOF horizontal coordinate (mm)	26.01 ± 1.34	25.75 ± 1.29	0.018
IOF vertical coordinate (mm)	33.86 ± 1.78	33.56 ± 1.94	0.003
IOF vertical diameter (mm)	3.29 ± 0.48	3.24 ± 0.47	0.468
IOF horizontal diameter (mm)	3.47 ± 0.25	3.50 ± 0.24	0.459
IOF–infraorbital margin distance (mm)	7.16 ± 0.86	7.28 ± 0.94	0.337
IOF–alveolar crest distance (mm)	30.67 ± 1.86	30.82 ± 1.97	0.516
Lateralization index	0.2035 ± 0.0075	0.2014 ± 0.0070	0.018
Vertical position index	0.5099 ± 0.0147	0.5144 ± 0.0152	0.003
Alveolo-orbital ratio	4.346 ± 0.565	4.307 ± 0.640	0.662

Paired Student’s *t*-test. Significant values are shown in bold.

### 3.4 Morphometric comparison according to AIOF presence (right)

Morphometric characteristics were further analyzed according to the presence of an accessory infraorbital foramen. On the right side, specimens with an AIOF exhibited a significantly smaller IOF vertical diameter (2.38 ± 0.28 mm vs. 3.48 ± 0.21 mm, *p* < 0.001) and a significantly shorter distance between the IOF and infraorbital margin (5.77 ± 0.43 mm vs. 7.45 ± 0.61 mm, *p* < 0.001) compared with specimens without an AIOF. In addition, the IOF–alveolar crest distance was significantly reduced (29.67 ± 1.85 mm vs. 30.89 ± 1.80 mm, *p* = 0.018), whereas the alveolo-orbital ratio was significantly higher (5.17 ± 0.55 vs. 4.17 ± 0.39, *p* < 0.001). No significant differences were observed in the horizontal coordinate, horizontal diameter, lateralization index, or vertical position index (Table 5).

**Table 5:** Comparison of morphometric characteristics according to the presence of accessory infraorbital foramen on the right side.

Parameter	AIOF absent (*n* = 85)	AIOF present (*n* = 18)	*p* value^*^
IOF horizontal coordinate (mm)	25.91 ± 1.32	26.49 ± 1.39	0.098
IOF vertical coordinate (mm)	33.71 ± 1.79	34.61 ± 1.62	0.046^†^
IOF vertical diameter (mm)	3.48 ± 0.21	2.38 ± 0.28	<0.001
IOF horizontal diameter (mm)	3.48 ± 0.25	3.43 ± 0.26	0.445
IOF–infraorbital margin distance (mm)	7.45 ± 0.61	5.77 ± 0.43	<0.001
IOF–alveolar crest distance (mm)	30.89 ± 1.80	29.67 ± 1.85	0.018^†^
Lateralization index	0.2033 ± 0.0075	0.2045 ± 0.0074	0.517
Vertical position index	0.5104 ± 0.0153	0.5073 ± 0.0113	0.409
Alveolo-orbital ratio	4.17 ± 0.39	5.17 ± 0.55	<0.001

^*^Independent samples *t*-test; ^†^Welch test.

### 3.5 Morphometric comparison according to AIOF presence (left)

Similarly, on the left side, specimens presenting an AIOF showed a significantly smaller IOF vertical diameter (2.41 ± 0.24 mm vs. 3.44 ± 0.24 mm, *p* < 0.001) and a significantly shorter IOF–infraorbital margin distance (5.76 ± 0.50 mm vs. 7.64 ± 0.60 mm, *p* < 0.001). The IOF–alveolar crest distance was also significantly lower (29.84 ± 2.26 mm vs. 31.05 ± 1.83 mm, *p* = 0.035), whereas the alveolo-orbital ratio was significantly greater (5.21 ± 0.63 vs. 4.09 ± 0.41, *p* < 0.001). In addition, the vertical position index was significantly lower in hemifaces with an accessory foramen (0.5077 ± 0.0103 vs. 0.5160 ± 0.0158, *p* = 0.006). No statistically significant differences were detected for the horizontal coordinate, horizontal diameter, or lateralization index (Table 6).

**Table 6:** Comparison of morphometric characteristics according to the presence of accessory infraorbital foramen on the left side.

Parameter	AIOF absent (*n* = 83)	AIOF present (*n* = 20)	*p* value^*^
IOF horizontal coordinate (mm)	25.83 ± 1.33	25.44 ± 1.11	0.231
IOF vertical coordinate (mm)	33.49 ± 2.02	33.85 ± 1.56	0.385^†^
IOF vertical diameter (mm)	3.44 ± 0.24	2.41 ± 0.24	<0.001
IOF horizontal diameter (mm)	3.51 ± 0.24	3.43 ± 0.23	0.187
IOF–infraorbital margin distance (mm)	7.64 ± 0.60	5.76 ± 0.50	<0.001
IOF–alveolar crest distance (mm)	31.05 ± 1.83	29.84 ± 2.26	0.035^†^
Lateralization index	0.2020 ± 0.0072	0.1990 ± 0.0058	0.058^†^
Vertical position index	0.5160 ± 0.0158	0.5077 ± 0.0103	0.006^†^
Alveolo-orbital ratio	4.09 ± 0.41	5.21 ± 0.63	<0.001

^*^Independent samples *t*-test, ^†^Welch test.

### 3.6 Sex-related differences

The comparison of morphometric parameters according to sex demonstrated significant sexual dimorphism in several craniofacial and infraorbital foramen measurements (Table 7). Male skulls exhibited significantly greater bizygomatic width (130.83 ± 1.24 vs. 125.06 ± 1.70 mm, *p* < 0.001) and nasion–anterior nasal spine height (71.68 ± 1.57 vs. 66.73 ± 1.35 mm, *p* < 0.001) than female skulls. Similarly, the mean horizontal (26.79 ± 0.81 vs. 25.06 ± 0.85 mm, *p* < 0.001) and vertical (35.39 ± 0.77 vs. 32.19 ± 0.82 mm, *p* < 0.001) coordinates of the infraorbital foramen were significantly greater in males, indicating a more laterally and superiorly positioned infraorbital foramen relative to the reference coordinate system. No significant sex-related differences were observed in the mean vertical diameter (*p* = 0.107), horizontal diameter (*p* = 0.841), infraorbital margin distance (*p* = 0.825), alveolar crest distance (*p* = 0.979), or alveolo-orbital ratio (*p* = 0.745). In contrast, both positional indices demonstrated significant differences between sexes. The lateralization index was significantly higher in males (0.2048 ± 0.0052 vs. 0.2004 ± 0.0057, *p* < 0.001), whereas the vertical position index was significantly higher in females (0.5176 ± 0.0094 vs. 0.5061 ± 0.0137, *p* < 0.001).

**Table 7:** Comparison of craniofacial and infraorbital foramen morphometric parameters according to sex.

Parameter	Male (*n* = 49)	Female (*n* = 54)	*p*
Bizygomatic width (mm)	130.83 ± 1.24	125.06 ± 1.70	<0.001
Nasion–ANS height (mm)	71.68 ± 1.57	66.73 ± 1.35	<0.001
Mean IOF_X (mm)	26.79 ± 0.81	25.06 ± 0.85	<0.001
Mean IOF_Y (mm)	35.39 ± 0.77	32.19 ± 0.82	<0.001
Mean IOF vertical diameter (mm)	3.21 ± 0.36	3.31 ± 0.28	0.107
Mean IOF horizontal diameter (mm)	3.49 ± 0.18	3.48 ± 0.19	0.841
Mean IOF–infraorbital margin distance (mm)	7.20 ± 0.63	7.23 ± 0.64	0.825
Mean IOF–alveolar crest distance (mm)	30.74 ± 1.52	30.75 ± 1.62	0.979
Mean lateralization index	0.2048 ± 0.0052	0.2004 ± 0.0057	<0.001
Mean vertical position index	0.5061 ± 0.0137	0.5176 ± 0.0094	<0.001
Mean alveolo-orbital ratio	4.34 ± 0.41	4.31 ± 0.39	0.745

Values are presented as mean ± standard deviation. Comparisons between sexes were performed using the independent samples *t*-test. Statistically significant *p* values are shown in bold.

### 3.7 Spatial density mapping

**Figure 2:**
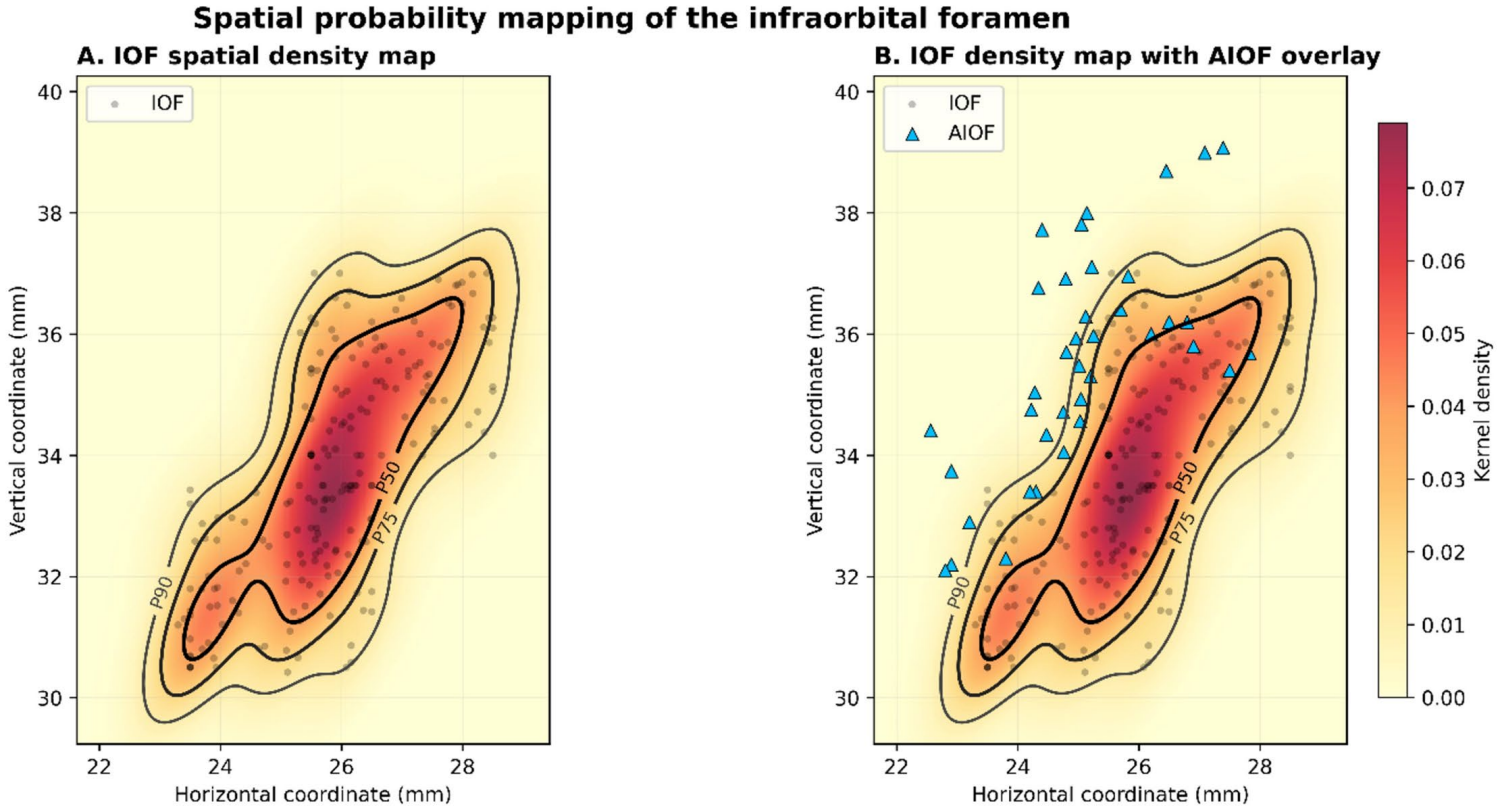
**(A)** Kernel density map showing the spatial distribution of the infraorbital foramen (IOF), with probability density contours (P50, P75, and P90). **(B)** Kernel density map of the IOF with accessory infraorbital foramina (AIOFs) superimposed, illustrating their spatial relationship to the principal IOF distribution.

Kernel density analysis demonstrated that the infraorbital foramina were concentrated within a relatively narrow anatomical region, with the highest density located centrally within the P50 contour. In contrast, accessory infraorbital foramina showed a wider and more heterogeneous spatial distribution surrounding the principal IOF cluster (Figure 2).

### 3.8 ROC analysis

**Figure 3:**
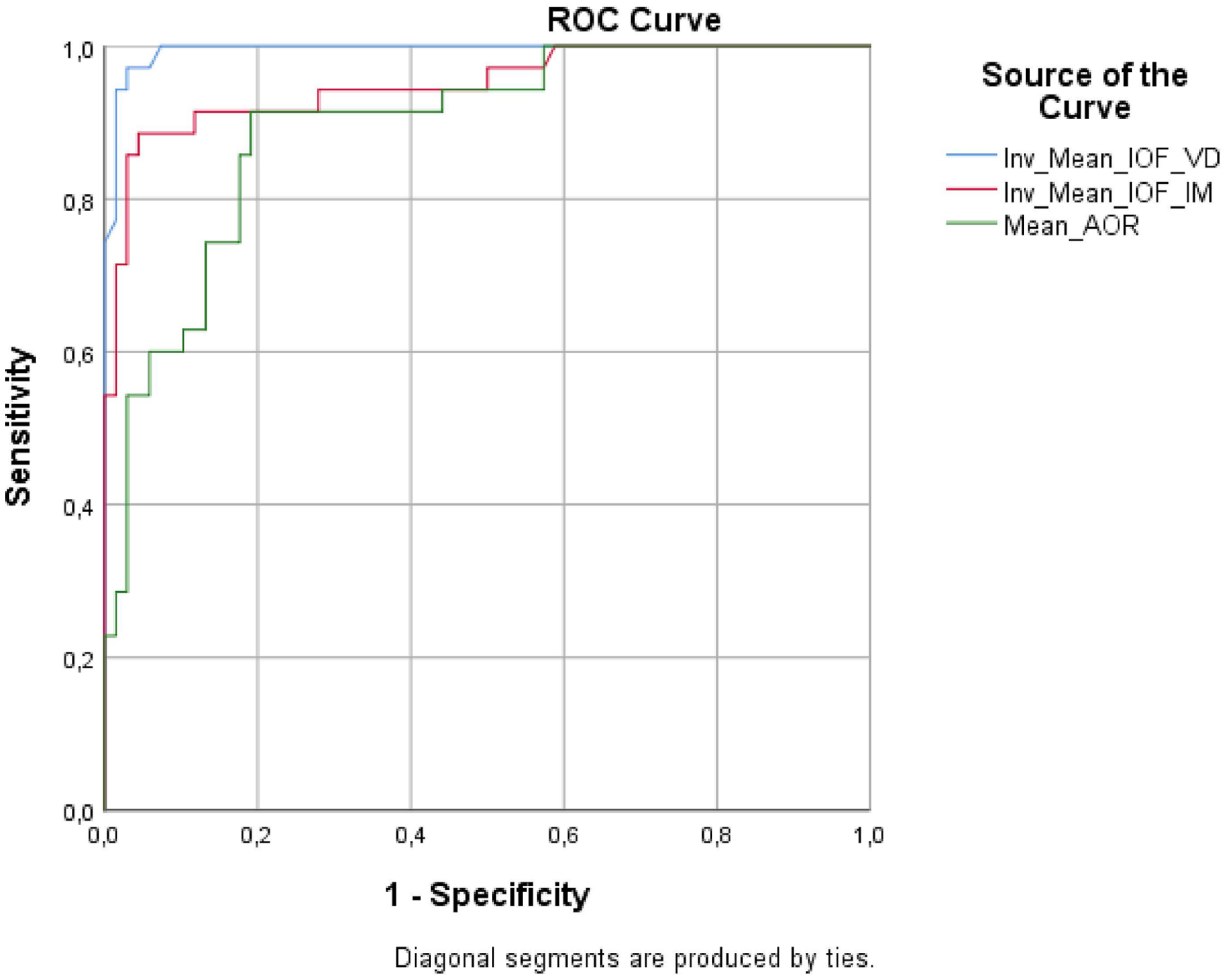
ROC curve analysis of morphometric parameters for predicting the presence of an accessory infraorbital foramen (AIOF).

ROC curves demonstrating the diagnostic performance of selected morphometric parameters associated with the presence of an accessory infraorbital foramen (Table 8; Figure 3). The inverse mean infraorbital foramen vertical diameter (Inv_Mean_IOF_VD) exhibited excellent discriminative ability (AUC = 0.995), followed by the inverse mean infraorbital margin distance (Inv_Mean_IOF_IM; AUC = 0.950) and the mean alveolo-orbital ratio (Mean_AOR; AUC = 0.894). These findings indicate that smaller infraorbital foramina and a shorter distance to the infraorbital margin, together with a higher alveolo-orbital ratio, are strongly associated with the presence of an accessory infraorbital foramen.

**Table 8:** ROC analysis of morphometric predictors of accessory infraorbital foramen.

Variable	AUC	95% CI	*p*
Inv_Mean_IOF_VD	0.995	0.986–1.000	<0.001
Inv_Mean_IOF_IM	0.950	0.903–0.997	<0.001
Mean_AOR	0.894	0.831–0.957	<0.001

### 3.9 Correlation analysis

Pearson correlation analysis demonstrated significant associations between craniofacial dimensions and infraorbital foramen localization parameters (Table 9). Bizygomatic width showed strong positive correlations with both horizontal (*r* = 0.834, *p* < 0.001) and vertical IOF coordinates (*r* = 0.917, *p* < 0.001). Similarly, upper facial height (Nasion–ANS height) was positively correlated with IOF_X (*r* = 0.771, *p* < 0.001) and IOF_Y (*r* = 0.870, *p* < 0.001). Among the positional indices, the lateralization index exhibited a very strong positive correlation with IOF_X (*r* = 0.872, *p* < 0.001), whereas the vertical position index demonstrated a strong negative correlation with IOF_Y (*r* = −0.640, *p* < 0.001). Bizygomatic width was positively associated with LI (*r* = 0.457, *p* < 0.001) and negatively associated with VPI (*r* = −0.462, *p* < 0.001). Regarding foraminal morphology, vertical diameter showed a strong positive correlation with the IOF–infraorbital margin distance (*r* = 0.672, *p* < 0.001) and a moderate negative correlation with the alveolo-orbital ratio (*r* = −0.588, *p* < 0.001). The strongest relationship observed in the dataset was the inverse correlation between IOF–infraorbital margin distance and alveolo-orbital ratio (*r* = −0.828, *p* < 0.001).

**Table 9:** Significant Pearson correlations between craniofacial dimensions and infraorbital foramen morphometric parameters.

Variable 1	Variable 2	*r*	*p*
Bizygomatic Width	Nasion–ANS Height	0.878	<0.001
Bizygomatic Width	Mean IOF_X	0.834	<0.001
Bizygomatic Width	Mean IOF_Y	0.917	<0.001
Bizygomatic Width	Mean LI	0.457	<0.001
Bizygomatic Width	Mean VPI	−0.462	<0.001
Nasion–ANS Height	Mean IOF_X	0.771	<0.001
Nasion–ANS Height	Mean IOF_Y	0.870	<0.001
Nasion–ANS Height	Mean LI	0.464	<0.001
Mean IOF_X	Mean IOF_Y	0.816	<0.001
Mean IOF_X	Mean LI	0.872	<0.001
Mean IOF_X	Mean VPI	−0.428	<0.001
Mean IOF_Y	Mean LI	0.501	<0.001
Mean IOF_Y	Mean VPI	−0.640	<0.001
Mean IOF_VD	Mean IOF_IM	0.672	<0.001
Mean IOF_VD	Mean IOF_AC	0.254	0.010
Mean IOF_VD	Mean AOR	−0.588	<0.001
Mean IOF_IM	Mean IOF_AC	0.336	0.001
Mean IOF_IM	Mean AOR	−0.828	<0.001
Mean IOF_AC	Mean LI	0.208	0.035
Mean IOF_AC	Mean AOR	0.230	0.019
Mean LI	Mean VPI	−0.280	0.004

IOF, infraorbital foramen; VD, vertical diameter; IM, infraorbital margin distance; AC, alveolar crest distance; LI, lateralization index; VPI, vertical position index; AOR, alveolo-orbital ratio.

## 4 Discussion

The present study provides a comprehensive morphometric and morphological evaluation of the IOF in adult dry skulls by integrating bilateral measurements, sex-based comparisons, morphometric indices, accessory infraorbital foramen assessment, correlation analyses, spatial density mapping, and ROC. Although numerous studies have investigated IOF anatomy, most have focused primarily on linear measurements and anatomical variations. Comparatively few studies have simultaneously examined bilateral symmetry, sexual dimorphism, proportional indices, and the relationship between IOF localization and craniofacial dimensions (19–22). Furthermore, quantitative spatial mapping and predictive analyses of accessory foramina remain rarely explored. Therefore, the present findings provide anatomically and clinically relevant reference data that may improve the accuracy of infraorbital nerve block procedures and surgical interventions involving the midfacial region. A comparative summary of IOF morphometric findings and AIOF prevalence reported in previous studies is provided in Supplementary Table S1.

The horizontal and vertical localization of the IOF observed in the present study was generally consistent with previous anatomical and radiological investigations. Kazkayasi et al. (23) demonstrated that the IOF occupies a relatively constant position with respect to surrounding facial landmarks despite individual anatomical variation, emphasizing its importance for infraorbital nerve block and orbital surgery. Similarly, Aggarwal et al. (24) reported a mean distance of approximately 6.3 mm between the IOF and infraorbital margin in North Indian skulls, while Varalakshmi et al. (25) described comparable measurements in South Indian specimens. The mean IOF-to-infraorbital margin distance observed in the present study (7.22 mm) falls within the range reported in previous investigations, supporting the concept that the IOF maintains a relatively predictable anatomical relationship with adjacent osseous landmarks despite population-specific variation.

One of the notable findings of the present study was the presence of statistically significant but quantitatively small right–left differences in IOF localization parameters, including horizontal and vertical coordinates as well as the lateralization and vertical position indices. Most previous dry-skull studies, including those by Potu et al. (26) and Varalakshmi et al. (25) reported no significant bilateral asymmetry, whereas more recent CT-based investigations have identified subtle side differences. Keleş et al. (27) demonstrated significant asymmetry in several IOF-related distances, particularly among female subjects, while Désiré et al. (28) also observed minor side-dependent variations despite limited clinical magnitude. In contrast, foraminal dimensions and most distance measurements did not differ significantly between sides in the present study, indicating that overall bilateral symmetry was largely preserved. These findings suggest that although bilateral symmetry is generally maintained, small positional differences may occur and should be considered during reconstructive procedures and image-guided interventions rather than assuming the contralateral side represents an exact anatomical template. The subtle bilateral differences in IOF position may reflect minor asymmetries in maxillary growth and remodeling rather than structural asymmetry of the foramen itself. This may explain why positional coordinates differed between sides while foraminal dimensions remained largely symmetrical (29, 30).

Sexual dimorphism represented another important aspect of the current study. Male skulls exhibited significantly greater bizygomatic width, upper facial height, horizontal and vertical IOF coordinates, and lateralization index, whereas females demonstrated significantly higher vertical position indices. In contrast, no significant sex-related differences were observed in IOF vertical diameter, horizontal diameter, IOF–infraorbital margin distance, IOF–alveolar crest distance, alveolo-orbital ratio, IOF shape, or orientation. These findings are largely consistent with previous investigations indicating that IOF position is influenced primarily by overall craniofacial dimensions, whereas the morphology of the foramen itself remains relatively stable between sexes. Keleş et al. (27) similarly reported larger IOF-related distances in males using CBCT analyses, whereas Thilakumara et al. (31) found no significant sex differences in a Sri Lankan population. Such discrepancies may reflect ethnic diversity, methodological differences, imaging modalities, and sample composition. The present findings further support the concept that craniofacial sexual dimorphism contributes substantially to the spatial localization of the IOF rather than to changes in foraminal morphology.

An important methodological contribution of this study is the evaluation of proportional morphometric indices, including the lateralization index, vertical position index, and alveolo-orbital ratio. Unlike absolute linear distances, these indices normalize IOF position relative to facial dimensions and may reduce variability attributable to skull size (32, 33). To date, most published studies have focused primarily on raw morphometric measurements, whereas standardized proportional indices remain largely unexplored. In the present study, several of these indices demonstrated significant bilateral and sex-related differences and showed meaningful associations with craniofacial dimensions, supporting their potential utility for describing IOF localization. Therefore, these parameters may facilitate more reliable comparisons among different populations and imaging modalities and could serve as useful reference values in future morphometric investigations.

Correlation analysis demonstrated that IOF localization is closely associated with craniofacial morphology. Particularly strong positive correlations were observed between bizygomatic width and both horizontal (*r* = 0.834) and vertical (*r* = 0.917) IOF coordinates, while upper facial height also showed strong associations with IOF position. In addition, the lateralization index exhibited a very strong correlation with horizontal IOF localization (*r* = 0.872). These findings indicate that enlargement of the facial skeleton is accompanied by proportional lateral and inferior displacement of the IOF (34). Similar observations were reported by Ceri and Ipek (35), who identified significant relationships between IOF position and facial height and width, while Keleş et al. (27) demonstrated a strong correlation between IOF-midline distance and adjacent maxillary landmarks. Together, these findings support the hypothesis that IOF position reflects overall craniofacial architecture rather than random anatomical variation. These correlations may reflect the coordinated developmental integration of the maxilla, orbit, and overall midfacial skeleton. The persistence of associations involving proportional indices further suggests that IOF localization is influenced not only by absolute craniofacial size but also by individual differences in facial proportions (2, 27, 36).

The presence of AIOF remains clinically important because accessory neurovascular branches may contribute to incomplete regional anesthesia and increase the risk of inadvertent neurovascular injury during surgical procedures (3, 5). In the present series, AIOFs were identified in approximately one-fifth of specimens, with no significant bilateral predominance. This prevalence is consistent with previous reports demonstrating considerable interpopulation variability, generally ranging from 10 to 30%. Canan et al. (37) emphasized the surgical relevance of accessory foramina in Turkish skulls, while Shin et al. (21), Şeker et al. (38), and Désiré et al. (28) reported comparable frequencies using cadaveric and radiological approaches. Developmentally, AIOFs may reflect variation in the branching and osseous passage of the infraorbital neurovascular bundle, with accessory branches emerging through separate bony canals. Anatomical studies have shown that accessory foramina may transmit branches of the infraorbital nerve, supporting their functional significance beyond being simple osseous variants. In this context, the smaller vertical diameter and shorter IOF–infraorbital margin distance observed in specimens with AIOFs may reflect an alternative pattern of neurovascular branching and bony canal organization. Clinically, such accessory pathways may contribute to incomplete infraorbital nerve blockade and increase the risk of neurovascular injury during midfacial procedures. However, these mechanisms remain hypothetical in the present dry-skull study because the associated soft-tissue structures could not be directly evaluated (21, 39–41).

Beyond prevalence data, the present study demonstrated that skulls with AIOFs exhibited significantly smaller vertical IOF diameters, shorter IOF–infraorbital margin distances, and higher alveolo-orbital ratios. ROC analysis further showed excellent discriminatory performance for vertical diameter and IOF–infraorbital margin distance, suggesting that these parameters may serve as useful anatomical indicators of AIOF presence. These findings indicate that accessory foramina are associated with distinct morphometric characteristics of the infraorbital region and may reflect underlying variations in neurovascular branching patterns, as suggested by previous cadaveric studies (21, 39, 40, 42). Recognition of such variations is particularly important during infraorbital nerve block, orbital floor reconstruction, endoscopic sinus surgery, facial trauma repair, and cosmetic midface procedures, where unrecognized accessory branches may contribute to persistent sensation or unexpected surgical complications.

The morphology and orientation of the IOF also have direct surgical implications. In the present study, round and oval foramina constituted the majority of IOF configurations, while horizontal, vertical, and oblique orientations were observed with varying frequencies. Previous studies have similarly demonstrated substantial variation in foraminal shape and canal orientation among different populations, with round and oval configurations generally predominating (1, 43). Such anatomical variability may influence the optimal angle and trajectory for infraorbital nerve block and should be considered during minimally invasive procedures involving the anterior maxilla, orbital floor, and midfacial region (9, 44, 45). The present findings reinforce the importance of individualized anatomical assessment. Beyond conventional morphometric measurements, the observed variability in IOF location, accessory foramina distribution, proportional indices, and bilateral asymmetry highlights the limitations of relying solely on generalized anatomical descriptions. Furthermore, the spatial density mapping performed in the present study demonstrated that although the IOF is concentrated within a relatively predictable anatomical region, substantial individual variation remains. Therefore, incorporation of population-specific morphometric data together with preoperative three-dimensional imaging may improve the accuracy of infraorbital nerve localization and reduce the risk of anesthetic failure or iatrogenic neurovascular injury during maxillofacial interventions.

Several limitations should be acknowledged. First, this investigation was performed on dry adult skulls, precluding assessment of soft tissue relationships that are directly relevant during clinical procedures. Second, although sex was estimated using established osteological methods, age at death and dental status could not be fully evaluated. Furthermore, because sex estimation was based on skeletal morphological characteristics rather than documented biological records, a degree of misclassification cannot be completely excluded. Third, the study was based on a single skeletal collection and therefore may not fully represent the anatomical diversity of broader populations. Nevertheless, the relatively large bilateral sample, comprehensive morphometric evaluation, assessment of sexual dimorphism and bilateral symmetry, inclusion of proportional indices, spatial density mapping, ROC-based analysis of accessory infraorbital foramina predictors, and detailed correlation analyses provide robust anatomical reference data with potential applications in regional anesthesia, maxillofacial surgery, reconstructive procedures, and anthropological research.

## 5 Conclusion

In conclusion, the present study demonstrates that infraorbital foramen localization is closely associated with craniofacial dimensions while exhibiting subtle bilateral asymmetry and significant sex-related differences in several morphometric parameters. The use of proportional morphometric indices, spatial density mapping, and accessory infraorbital foramen analysis provided additional insights into the anatomical variability of the infraorbital region. Furthermore, ROC analysis identified vertical IOF diameter and IOF–infraorbital margin distance as strong morphometric indicators associated with the presence of accessory infraorbital foramina. Collectively, these findings provide clinically relevant anatomical reference data that may contribute to improved preoperative planning and safer performance of infraorbital nerve block, orbital reconstruction, facial trauma surgery, and other procedures involving the midfacial skeleton.

## Data Availability

The raw data supporting the conclusions of this article will be made available by the authors, without undue reservation.
