# Supplementary material for: Morphometric analysis and spatial localization of the infraorbital foramen in adult dry human skulls: bilateral symmetry, sexual dimorphism, and accessory foramina
Source: Front Med (Lausanne). 2026 Aug 5;13:1914818. doi: 10.3389/fmed.2026.1914818 (PMC13485796; doi:10.3389/fmed.2026.1914818)
Supplement: Supplementary file 1 [file Table_1.DOCX]

Supplementary Table S1. Comparative summary of infraorbital foramen morphometry and accessory infraorbital foramen prevalence in previous studies and the present study.

| **Study** | **Year** | **Population/Country** | **Method** | **Sample size** | **IOF-IOM distance** | **Other comparable IOF measurements** | **AIOF prevalence** | **Side-related findings** | **Sex-related findings** |
| --- | --- | --- | --- | --- | --- | --- | --- | --- | --- |
| Kazkayasi et al. | 2001 | Turkish adult bony heads | Dry skull + cephalometry | 35 skulls / 70 sides | 7.19 ± 1.39 mm | IOF-lateral nasal border: 17.23 ± 2.64 mm; IOF-canine tooth: 33.94 ± 3.15 mm; IOC: 22.95 ± 5.43 mm; IOG: 5.95 ± 4.90 mm | Double IOF/AIOF: 4/70 sides = 5.7%; bilateral in 1 skull | NR | Sex not determined |
| Canan et al. | 1999 | Turkish cadavers/crania | Cadaveric dissection + skeletal material | IOF localization: 45 cadavers; AIOF: 119 crania and 229 maxillae, total 467 IOF specimens | Men: 10.9 mm; women: 8.3 mm | IOF on defined vertical line: R 34/45 = 75.6%; L 31/45 = 68.9% | Single AIOF: 54/467 = 11.5%; double AIOF: 6/467 = 1.28% | Single AIOF: R 28, L 26; no side difference reported | IOF closer to IOM in women than men; AIOF sex analysis NR |
| Agthong et al. | 2005 | Asian/Thai adult skulls | Dry skull | 110 crania; 70 male, 40 female | R: 0.78 ± 0.02 cm; L: 0.80 ± 0.02 cm | IOF-ANS R/L: 3.41 ± 0.02 / 3.43 ± 0.02 cm; IOF angle R/L: 25.1 ± 0.4° / 26.8 ± 0.4° | Double IOF/AIOF: R 4/110 = 3.6%; L 5/110 = 4.5% | IOF angle differed significantly between sides | IOF-ANS significantly smaller in females; IOF-IOM not significantly different by sex |
| Aggarwal et al. | 2015 | Indian adult dry skulls | Dry skull | 67 skulls / 133 hemi-skulls | Overall: 6.33 ± 1.39 mm; R: 6.37 ± 1.52 mm; L: 6.28 ± 1.25 mm | IOF-ML: 25.69 ± 2.37 mm; IOF-PA: 15.19 ± 1.70 mm; IOF-AB: 28.41 ± 2.82 mm; transverse diameter: 2.72 ± 0.95 mm; vertical diameter: 3.54 ± 1.11 mm | 28/133 hemi-skulls = 21%; bilateral in 4/67 skulls; double accessory foramina in 1 case | No significant right-left differences | Sex records unavailable |
| Gibelli et al. | 2019 | Italian Caucasoid contemporary skeletal collection | Dry skull | 100 skulls; 50 male, 50 female | R: 6.6 ± 1.7 mm; L: 6.7 ± 1.6 mm | IOF-ANS R/L: 36.3 ± 2.8 / 36.6 ± 2.6 mm; angle R/L: 37.1 ± 5.2° / 35.3 ± 4.6° | 24% of crania; R only 6%, L only 10%, bilateral 8% | Angle differed by side; IOF-IOM did not | IOF-ANS larger in males; AIOF: 32% males, 16% females, not significant |
| Ceri & Ipek | 2020 | Turkish dry skulls | Dry skull | 75 skulls | R: 6.75 ± 1.54 mm; L: 6.32 ± 1.50 mm | FOI-ANS R/L: 32.98 ± 2.48 / 33.88 ± 3.36 mm; FOI-piriform aperture R/L: 15.65 ± 2.19 / 16.61 ± 2.64 mm | NR | Side-specific measurements reported | Sex NR |
| Potu et al. | 2020 | South Indian adult dry skulls | Dry skull | 14 skulls / 28 sides | R: 5.49 ± 1.10 mm; L: 5.85 ± 1.06 mm | Vertical diameter R/L: 3.95 ± 0.86 / 3.82 ± 0.83 mm; transverse diameter R/L: 3.70 ± 1.20 / 3.86 ± 1.54 mm; IOF-ANS R/L: 33.62 ± 2.22 / 33.13 ± 2.30 mm | Reported as 14.2% on right and 14.2% on left; raw numerator NR | No significant right-left differences | Sex/age unknown |
| Thilakumara et al. | 2021 | Sri Lankan adults | CBCT | 61 scans / 122 IOFs | R: 5.56 ± 3.95 mm; L: 4.91 ± 2.08 mm | IOF-LNW R/L: 11.96 ± 3.45 / 12.18 ± 3.35 mm; IOF-MM R/L: 27.13 ± 2.60 / 26.99 ± 2.73 mm | R: 1.6%; L: 4.9% of sides | No significant side differences | No significant sex differences |
| Varalakshmi & Nayak | 2021 | South Indian adult dry skulls | Dry skull | 90 skulls | R: 6.34 ± 1.63 mm; L: 6.43 ± 1.48 mm | Vertical diameter R/L: 3.29 ± 0.82 / 3.37 ± 0.81 mm; horizontal diameter R/L: 3.77 ± 0.85 / 3.90 ± 0.96 mm | 7/90 skulls = 7.7%; bilateral in 1 skull; unilateral in 6 skulls (2 right, 4 left) | Side-specific values reported | Sex NR |
| Suntiruamjairucksa & Chentanez | 2022 | Thai dry skulls | Dry skull; cadaveric validation | 216 skulls / 432 sides; 15 cadavers for validation | NR | IOF vertical distance to ANS-Z line: 15.14 ± 1.99 mm; IOF D:A ratio: 63.35 ± 3.90% | 86/432 sides = 19.91%; 42 skulls with AIOF, 21 bilateral; male 60/306 sides, female 26/126 sides | Left 46 AIOFs, right 40 AIOFs; no significant side difference in predictive distances | No significant sex difference in predictive distances; AIOF side prevalence similar by sex |
| Shin et al. | 2020 | Korean cadavers | Cadaveric dissection | 25 cadavers / 44 hemifaces | NR | AIOF-IOF distance: 7.2 mm; AIOF mostly superomedial to IOF | 8/44 hemifaces = 18.2% | 5 right, 3 left; no laterality difference | Sex-stratified findings NR |
| Désiré et al. | 2023 | Cameroonian adults | CT | 208 CT scans / 416 IOFs | R: 6.87 ± 1.87 mm; L: 7.17 ± 1.91 mm | Transverse diameter R/L: 2.10 ± 0.52 / 1.97 ± 0.51 mm; IOF-MFL R/L: 28.7 ± 3.23 / 27.4 ± 4.42 mm | R: 51/208 subjects = 24.5%; L: 62/208 subjects = 29.8%; overall subject prevalence NR | Side-specific AIOF values reported; bilateral distribution NR | Sex-specific AIOF/morphometric values NR |
| An et al. | 2023 | Thai adults | CBCT | 507 patients | NR | AIOF-IOF distance R/L: 5.12 ± 2.08 / 5.13 ± 1.84 mm; AIOF diameter R/L: 0.69 ± 0.18 / 0.80 ± 0.29 mm | 36/507 patients = 7.1%; unilateral 22/36, bilateral 12/36; double AIOF in 2 patients | No significant side association | No significant sex association |
| Şeker et al. | 2025 | Turkish adolescents and adults | CBCT | 315 patients / 630 sides | R: 8.44 ± 1.81 mm; L: 8.39 ± 1.73 mm | Horizontal length R/L: 3.87 ± 0.55 / 3.86 ± 0.59 mm; vertical length R/L: 4.20 ± 0.56 / 4.24 ± 0.65 mm; IOF-ML R/L: 26.50 ± 2.71 / 26.82 ± 2.64 mm | 199/630 sides = 31.6%; patient-level prevalence NR | AIOF: R 96, L 103; IOF-ML age difference on right | IOF dimensions larger in males; AIOF more frequent in males on right |
| Keleş et al. | 2026 | Turkish adults | 3D CT | 316 individuals / 632 IOFs | Overall: 8.32 ± 1.70 mm | VIOF: 2.55 ± 0.41 mm; TIOF: 2.44 ± 0.41 mm; IOF-PA: 14.02 ± 1.84 mm; IOF-ML: 24.41 ± 2.29 mm; IOF-AB: 31.43 ± 2.89 mm | NR | Side differences in females for IOF-IOM, IOF-ML, IOF-AB; in males for IOF-ML | Most parameters larger in males except IOF-IOM |
| Present study | 2026 | Turkish adult dry human skulls | Dry skull | 103 skulls / 206 hemimaxillae | Overall: 7.22 ± 0.90 mm; R: 7.16 ± 0.86 mm; L: 7.28 ± 0.94 mm | IOF horizontal coordinate overall/R/L: 25.88 ± 1.32 / 26.01 ± 1.34 / 25.75 ± 1.29 mm; IOF vertical coordinate overall/R/L: 33.71 ± 1.86 / 33.86 ± 1.78 / 33.56 ± 1.94 mm; vertical diameter: 3.27 ± 0.48 mm; horizontal diameter: 3.49 ± 0.24 mm | 38/206 hemimaxillae = 18.4% | Significant bilateral differences in IOF horizontal and vertical coordinates | Significant sex-related differences in craniofacial dimensions and IOF position |

Footnotes: IOF, infraorbital foramen; AIOF, accessory infraorbital foramen; IOM, infraorbital margin; R, right; L, left; NR, not reported in the examined full text; ANS, anterior nasal spine; ML/MFL/MM, midline/midfacial or maxillary midline as defined by each study. Values are reported as published in the original full text. AIOF prevalence denominators differ across studies and may refer to skulls/patients, sides/hemifaces, or foramina/specimens; denominator is stated in the table wherever verifiable.
